# Thiols as a marker of inflammatory bowel disease activity: a systematic review

**DOI:** 10.1186/s12876-023-02711-9

**Published:** 2023-03-28

**Authors:** Rebeca Araujo Passos, Priscila Ribas Farias Costa, Claudia Feio da Maia Lima, George Mariane Soares Santana, Victor David, Geisa de Jesus Santos, Cyrla Zaltman, Marcia Soares-Mota, Raquel Rocha

**Affiliations:** 1 Postgraduate Program in Food, Nutrition and Health. School of Nutrition, Federal University of Bahia, Bahia, Brazil; 2 Health Sciences Center, Federal University of Recôncavo da Bahia (UFRB), Bahia, Brazil; 3 Federal University of Rio de Janeiro (UFRJ), Bahia, Brazil

**Keywords:** Inflammatory Bowel Disease, Crohn's Disease, Ulcerative Colitis, Biomarkers, Thiols, Systematic Review

## Abstract

**Background:**

Evidence indicates that inflammation in Inflammatory Bowel Disease (IBD) is associated with increased systemic levels of reactive oxygen species. Systemic oxidative stress has been associated with reduced levels of plasma thiols. Less invasive tests capable of reflecting and predicting IBD activity are increasingly sought after. We sought to systematically review the evidence inherent in serum thiol levels as a marker of Crohn's Disease and Ulcerative Colitis activity (PROSPERO: CRD42021255521).

**Methods:**

The highest quality documents for systematic reviews standards were used as reference. Articles were searched on Medline via PubMed, VHL, LILACS, WOS, EMBASE, SCOPUS, COCHRANE, CINAHL, OVID, CTGOV, WHO/ICTRP, OPENGREY, BDTD and CAPES, between August, 03 and September, 03 on 2021. Descriptors were defined according to the Medical Subject Heading. Of the 11 articles selected for full reading, 8 were included in the review. It was not possible to perform a pooled analysis of the studies, as there were no combinable studies between subjects with active IBD and controls/inactive disease.

**Results:**

Findings from the individual studies included in this review suggest an association between disease activity and systemic oxidation, as measured by serum thiol levels, however, there are limitations that preclude weighting the study results in a meta-analysis.

**Conclusions:**

We recommend conducting better-designed and controlled studies, that include individuals of both phenotypes and at different stages of IBD, involving a larger number of participants, using the standardization of the technique for measuring serum thiols, to confirm whether thiols can be a good parameter for monitoring the clinical course of these intestinal diseases and the degree of clinical applicability.

**Supplementary Information:**

The online version contains supplementary material available at 10.1186/s12876-023-02711-9.

## Introduction

Inflammatory Bowel Disease (IBD) has two most common forms of presentation: Ulcerative Colitis (UC) and Crohn's Disease (CD), which are characterized by chronic inflammatory processes [1–3], with alternating remission periods and disease activity. The various therapies aim to keep patients as long as possible without clinical manifestations. Predicting exacerbations of inflammation is challenging due to the highly variable spectrum of symptomatology and lack of availability of IBD-sensitive biomarkers [2].

Evidence suggests that inflammation in IBD is associated with increased systemic levels of reactive oxygen species (ROS), which cause oxidative stress, reducing antioxidants and favoring the inflammatory environment; however, there is a need to identify biomarkers capable of predicting IBD activity [1, 2, 4–9].

The reduction in free thiol groups in plasma proteins (plasma thiols) reflects a condition of systemic oxidative stress, since thiols are primary substrates for ROS. Plasma thiols are considered a robust measure of overall redox status in vivo, considering that thiol groups are rapidly oxidized by ROS metabolites, and therefore systemic oxidative stress is associated with reduced plasma thiol levels. Therefore, plasma thiol levels could be considered predictors for monitoring disease activity and inflammatory degree in IBD [1, 2, 4–10].

Endoscopic evaluation is the gold standard for diagnosing and verifying IBD activity. However, due to its invasive nature and the need for bowel preparation, it is poorly accepted by individuals with UC and CD. Additionally, it is an expensive, time-consuming and risky procedure. In clinical practice, the use of clinical IBD indices are commonly applied to quantify patient-reported disease activity, although these indices are inaccurate to reflect mucosal inflammation [2].

In this perspective, less invasive tests capable of reflecting and predicting IBD activity are increasingly sought after. Fecal calprotectin is currently considered an important biomarker - capable of detecting inflammatory factors related to disease activity; however, both its diagnostic accuracy to differentiate degrees of disease activity and its clinical applicability demand further studies [2, 11].

Searches in this area of investigation are incessant, and aim to identify substitute markers for conventionally used markers, which have the potential to monitor disease activity and to detect early IBD exacerbations.

We sought to systematically review the evidence inherent in the measurement of serum thiol levels as a marker of IBD activity, the potential for association to predict IBD activity and the degree of clinical applicability for managing IBD.

## Methods

### Protocol and registration

To carry out the study, the Preferred Reporting Items for Systematic Reviews and Meta-Analyses (PRISMA) guidelines strategy, composed of 27 items [12, 13] and the recommendations of the Cochrane Collaboration [14, 15]) were used as reporting standards. The study was registered in the PROSPERO Protocol database (York University) under registration number CRD42021255521.

### Sources of information and search strategies

Two independent reviewers (R.A.P. and G.M.S.S.) performed a literature review following the selection criteria for the studies, in electronic databases, in the trial registry databases and gray literature (Table 1). The searches were conducted using Medline via PubMed, VHL, LILACS, WOS, EMBASE, SCOPUS, COCHRANE, CINAHL, OVID, CTGOV, WHO/ICTRP, OPENGREY, BDTD and CAPES. The research question, the controlled vocabulary and the keywords used in the search strategies were structured by the acronym PECOS: Population: Individuals with IBD (UC or CD); Exposure: Measurement of free thiols in serum; Comparison: Disease activity assessment methods (clinical, endoscopic and histological); Outcomes primary outcomes/outcomes): IBD activity markers; Study design: Observational and interventional studies that correlated markers of IBD activity with plasma thiol levels. Only the terms for the P (population) and E (exposure) components were defined, to avoid undesirable specifications in the data search. The search strategy was designed following the guidance of an expert librarian and according to the specificity of each database, whenever possible, using the controlled vocabulary of subject descriptors (Mesh / Medline and DeCs / VHL). Disagreements between the researchers who retrieved the data were resolved by consensus.

**Table 1:** Sources of information/ electronic databases and search strategies medline

#1 (Inflammatory Bowel Diseases [MESH] OR Inflammatory Bowel Disease [TIAB] OR Bowel Diseases Inflammatory [TIAB] OR Crohn Disease [MESH] OR Crohn Disease [TIAB] OR Crohn's Disease [TIAB] OR Ulcerative Colitis [TIAB] OR Idiopathic Proctocolitis [TIAB] OR Proctocolitis [TIAB])	#2 (Sulfhydryl Compounds [MESH] OR Compounds Sulfhydryl [TIAB] OR Sulfhydryl Compound [TIAB] OR Thiol [TIAB] OR Thiol* [TIAB])
#1 AND #2	
**PMC**	
#1 (Inflammatory Bowel Diseases [MESH] OR Inflammatory Bowel Disease [Title] OR Crohn Disease [MESH] OR Crohn Disease [Title] OR Crohn's Disease [Title] OR Ulcerative Colitis [Title] OR Proctocolitis [Title])	#2 (Sulfhydryl Compounds [MESH] OR Sulfhydryl Compound [Title] OR Thiol [Title] )
#1 AND #2	
**BVS**	
#1 (mh: "Doenças Inflamatórias Intestinais" OR mh: "Inflammatory Bowel Diseases" OR tw: "Doenças Inflamatórias Intestinais" OR tw: "Inflammatory Bowel Diseases" OR tw: "Doenças Inflamatórias do Intestino" OR mh: "Doença de Crohn" OR tw: "Doença de Crohn" OR mh: "Crohn Disease" OR tw: "Crohn Disease" OR mh: "Colitis, Ulcerative" OR tw: "Colitis Ulcerative" OR mh: "Colite Ulcerativa" OR tw: "Colite Ulcerativa")	#2 (mh: "Compostos de Sulfidrila" OR tw: "Compostos de Sulfidrila" OR mh: "Sulfhydryl Compounds" OR tw: "Sulfhydryl Compounds" OR tw:"Thiol")
#1 AND #2	
**LILACS**	
#1 (mh: "Doenças Inflamatórias Intestinais" OR mh: "Inflammatory Bowel Diseases" OR tw: "Doenças Inflamatórias Intestinais" OR tw: "Inflammatory Bowel Diseases" OR tw: "Doenças Inflamatórias do Intestino" OR mh: "Doença de Crohn" OR tw: "Doença de Crohn" OR mh: "Crohn Disease" OR tw: "Crohn Disease" OR mh: "Colitis, Ulcerative" OR tw: "Colitis Ulcerative" OR mh: "Colite Ulcerativa" OR tw: "Colite Ulcerativa")	#2 (mh: "Compostos de Sulfidrila" OR tw: "Compostos de Sulfidrila" OR mh: "Sulfhydryl Compounds" OR tw: "Sulfhydryl Compounds" OR tw:"Thiol")
#1 AND #2	
**SCOPUS**	
#1 TITLE-ABS-KEY ( "inflammatory bowel diseases" OR "inflammatory bowel disease" OR "bowel diseases inflammatory" OR "crohn disease" OR "crohn's disease" OR "ulcerative colitis" OR "idiopathic proctocolitis" OR "proctocolitis" )	#2 TITLE-ABS-KEY ( "Sulfhydryl Compounds" OR "Compounds Sulfhydryl" OR "Sulfhydryl Compound" OR thiol* )
#1 AND #2	
**WOS**	
#1 ALL= ("inflammatory bowel diseases" OR "inflammatory bowel disease" OR "bowel diseases inflammatory" OR "crohn disease" OR "crohn's disease" OR "ulcerative colitis" OR "idiopathic proctocolitis" OR "proctocolitis")	#2 ALL= ("Sulfhydryl Compounds" OR "Compounds Sulfhydryl" OR "Sulfhydryl Compound" OR thiol*)
#1 AND #2	
**EMBASE**	
#1 'inflammatory bowel diseases'/exp OR 'inflammatory bowel diseases' OR 'crohn disease'/exp OR 'crohn disease' OR 'ulcerative colitis'/exp OR 'ulcerative colitis' OR 'idiopathic proctocolitis' OR 'proctocolitis'/exp OR 'proctocolitis'	#2 'inflammatory bowel diseases'/exp OR 'inflammatory bowel diseases' OR 'crohn disease'/exp OR 'crohn disease' OR 'ulcerative colitis'/exp OR 'ulcerative colitis' OR 'idiopathic proctocolitis' OR 'proctocolitis'/exp OR 'proctocolitis'
#1 AND #2	
**CINAHL**	
#1 (Inflammatory Bowel Diseases [MESH] OR Inflammatory Bowel Disease [TIAB] OR Bowel Diseases Inflammatory [TIAB] OR Crohn Disease [MESH] OR Crohn Disease [TIAB] OR Crohn's Disease [TIAB] OR Ulcerative Colitis [TIAB] OR Idiopathic Proctocolitis [TIAB] OR Proctocolitis [TIAB])	#2 (Sulfhydryl Compounds [MESH] OR Compounds Sulfhydryl [TIAB] OR Sulfhydryl Compound [TIAB] OR Thiol [TIAB] OR Thiol* [TIAB])
#1 AND #2	
**COCHRANE**	
#1 MeSH descriptor: [Inflammatory Bowel Diseases] explode all trees	#2 Inflammatory Bowel Disease OR Bowel Diseases Inflammatory
#3	#1 OR #2
#4 MeSH descriptor: [Crohn Disease] explode all trees	#5 Crohn Disease OR Crohn's Disease OR Ulcerative Colitis OR Idiopathic Proctocolitis OR Proctocolitis
#6	#4 OR #5
#7	#3 OR #6
#8 MeSH descriptor: [Sulfhydryl Compounds] explode all trees	#9 Compounds Sulfhydryl OR Sulfhydryl Compound OR Thiol*
#10	#8 OR #9
#11	#7 AND #10
**OVID**	
(Inflammatory Bowel Diseases and Sulfhydryl Compounds).af.	
**CTGOV**	
Inflammatory Bowel Disease OR Bowel Diseases Inflammatory OR Crohn Disease OR Crohn's Disease OR Ulcerative Colitis OR Idiopathic Proctocolitis OR Proctocolitis Compounds | Sulfhydryl OR Sulfhydryl Compound OR Thiol*	
**WHO**	
Inflammatory Bowel Disease OR Bowel Diseases Inflammatory OR Crohn Disease OR Crohn's Disease OR Ulcerative Colitis OR Idiopathic Proctocolitis OR Proctocolitis Compounds | Sulfhydryl OR Sulfhydryl Compound OR Thiol*	
**OPENGREY**	
#1 ("inflammatory bowel diseases" OR "inflammatory bowel disease" OR "bowel diseases inflammatory" OR "crohn disease" OR "crohn's disease" OR "ulcerative colitis" OR "idiopathic proctocolitis" OR "proctocolitis")	
**BDTD**	
(Inflammatory Bowel Diseases and Sulfhydryl Compounds)	
**ICTRP**	
(Inflammatory Bowel Diseases and Sulfhydryl Compounds)	
**CAPES**	
("inflammatory bowel diseases" OR "inflammatory bowel disease" OR "bowel diseases inflammatory" OR "crohn disease" OR "crohn's disease" OR "ulcerative colitis" OR "idiopathic proctocolitis" OR "proctocolitis") AND ("Sulfhydryl Compounds" OR "Compounds Sulfhydryl" OR "Sulfhydryl Compound" OR thiol*)	

The searches were carried out between August, 03 and September, 03 on 2021. No date or language limits were imposed on the search, and no search filters were used. All databases were periodically monitored until the completion of the study.

### Eligibility criteria

Studies that met the following criteria were included: (I) intervention (clinical or community trial, randomized or non-randomized) and observational studies; (II) that had a population consisting of individuals diagnosed with IBD (UC or CD), based on clinical and/or endoscopic criteria, of any sex and any age group and at any stage of the disease; (III) who used serum free thiol measurements to assess the oxidative status/inflammatory bowel activity; (IV) who used classical disease activity indices/methods (clinical and/or histological) associated with thiol dosage as an outcome. Review studies, literature reviews, systematic reviews, meta-analyses, descriptive studies such as case reports, case studies and case series were excluded. Studies conducted in animals and in-vitro studies were also excluded. We did not include studies that evaluated individuals with comorbidities/chronic diseases unrelated to the underlying disease (nephropathies, liver diseases, chronic pancreatitis, diabetes mellitus, gallstones, hematologic changes).

### Selection of studies

Initially, the articles were selected by title and abstract and those that did not meet the eligibility criteria were excluded. Articles contained in more than one database were considered only once, using Rayyan bibliography management software [16] to exclude duplicate articles. Full articles were read when there was not enough information in the title and abstract to make an accurate decision about inclusion or exclusion from the study. One study had a title relevant to the search, but no abstract was available; the article’s author was contacted by e-mail and sent the article for reading. However, this study did not meet the eligibility criteria and was therefore excluded.

At this stage, the reason for exclusion was recorded in Rayyan [16] to compose the study selection flow.

Disagreements between the two authors were resolved in a consensus meeting, after opening the shielding of decisions between the reviewers and identifying conflicting studies, which was performed using the Rayyan software [16].

The eligible studies went through the complete reading stage, by two independent reviewers (R.A.P. and G.M.S.S.). These documents were evaluated according to the previously defined eligibility criteria. To framed better literature saturation, the reference lists of included studies and relevant reviews, manually identified through the search, were analyzed to make it possible to add studies that were not indexed in the databases, but that were relevant for inclusion in this review; however, no other studies were found.

**Fig. 1:**
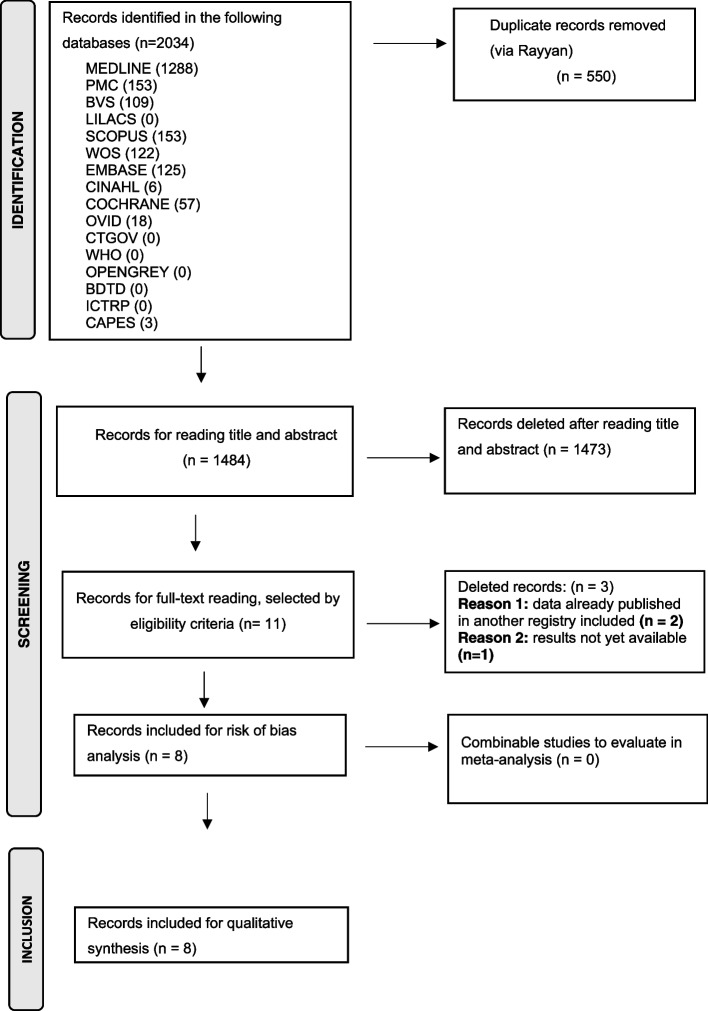
Prisma (preferred reporting items for systematic reviews and meta-analyses) flowchart of selected articles and selection process for systematic review and meta-analysis [13]

The Figure 1 summarizes the study selection process.

### Data extraction

The original records included in the final list were read in full and the information contained therein was recorded in an Excel® spreadsheet, prepared by one author (R.A.P.), reviewed and approved by a second reviewer (C.F.M.L.) standardized to assess the quality and synthesize the evidence.

### Assessment of methodological quality

The assessment of the risk of bias and effectiveness and safety estimates in of the included studies was performed by two independent investigators (R.A.P. and V.D.), using: I. the Newcastle-Ottawa Checklist for observational, non-randomized, case-control studies (Table 2); II. the ROBINS-I (Risk Of Bias In Non-randomised Studies – of Interventions) (Table 3) for the non-randomized intervention studies [17].

**Table 2:** Assessment of risk of bias of studies and quality according to the newcastle otawa scale

**Autor/ Ano**	**Selection**				**Comparability**		**Outcome**		**Total**
	Adequate definition	Representativeness of the cases	Selection of controls	Definition of controls	Comparability of cases and controls on the basis of the design or analysis	Ascertainment of exposure	Same method of ascertainment for cases and controls	Non-Response rate	9 ★
Baskol et al., 2008 [4]	★	★	-	★	★ ★	★	★	-	7/9
Ankinci et al., 2016 [5]	★	★	-	-	★ ★	★	★	-	6/9
Yuksel et al., 2016 [6]	★	★	★	★	-	★	★	-	6/9
Neselioglu et al., 2018 [7]	★	★	★	★	★	★	★	-	7/9
Bourgonje et al., 2019 [1]	★	★	★	★	★ ★	★	★	-	8/9
Bourgonje et al., 2019 [2]	★	★	★	★	★ ★	★	★	-	8/9
Neubauer et al., 2019 [8]	★	★	-	★	★ ★	★	★	-	7/9

**Table 3:**
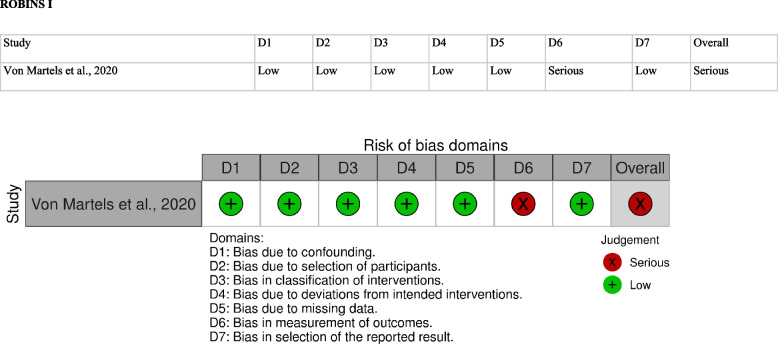
Assessment of risk of bias of studies and quality according to the robins I

### Statistical analysis

The present study adopted thiol levels as the outcome variable and UC and CD as exposure variables. Considering that the meta-analysis is the pooled analysis of two or more combinable studies [18, 19], after analyzing the data from the included studies, limitations were identified that prevented the weighting of the results of the studies in a meta-analysis. It was impossible to perform the meta-analysis for the inactive/remission of diseases and the different types of thiol (total and native) due to the absence of combinable studies.

## Results

### Search results

According to the search strategy (see PRISMA diagram, Figure 1), 2034 articles were found in the accessed databases. Of these, 550 articles were excluded after database screening and removal of duplicates and 1484 studies were read for titles and abstracts. Analyzing titles and abstracts resulted in the exclusion of 1473 studies for not meeting the eligibility criteria. The remaining eleven (11) records were read in full and three (3) of them were excluded for the following reasons: two (2) records had data published in poster format, which had data from two other included studies in this review, being excluded, to avoid duplication of data analysis. One (1) study published in trial format was excluded, as there were no results available; there was an attempt to contact the authors of this study to obtain information on partial data, unpublished or in preprint, however, there was no response to email.

### Description of articles included in the systematic review

The included studies in this review were developed in the following countries: Turkey, Netherlands and Poland. The Table 4 shows the summary of the characteristics and main results of the studies. In total, 979 subjects were evaluated: 321 with UC, 342 with CD and 316 healthy controls participated in the studies. Only two studies [1, 7] exclusively evaluated subjects in remission (one study evaluated 20 subjects with UC in remission and the other evaluated 51 subjects with CD in remission, respectively) and only one study exclusively evaluated individuals with active CD [25]. The included studies in this review had data collected between 2006 and 2018 and were published between 2008 and 2020. There was no standardization regarding the type of thiol measured as an expression of the oxidative state nor the method used for its dosage. Of the included studies, five (1, 2, 4, 8, 25) used the method described by Elman´s et al. [20] for thiol measurement, but only four [1, 2, 4, 25] corrected for plasma albumin, as described by Hu et al. [21] and Turel et al. [22]. The method proposed by Erel & Neselioglu [23] was used for thiol dosage in one study [6]. The other two studies [5, 7] included in this review did not explain the reagent used to measure thiols. All studies described the use of spectrophotometric analysis for thiol dosage.

**Table 4:** Synthesis of studies included in the systematic review of the association between the inflammatory bowel disease and thióis

FIRST AUTHOR	YEAR	COUNTRY	CHARACTERIZATION OF THE POPULATION (PHENOTYPE AND DISEASE PHASE)	N DISEASE	N CONTROL HEALTHY	OUTCOME	RESULTS
Baskol et al. [4]	2008	Turkey	UC active	UC: 30	30	Serum tiol total	significantly serum tiol total was increased in UC group compared with HC
Akinci et al. [5]	2016	Turkey	UC: active and inactive CD: active and inactive	UC active: 16 UC inactive: 43 CD active: 49 CD inactive: 69	30	Serum thiol (native and total)	significantly higher native and total thiol in CD inactive compared to CD active significantly native thiol were higher in the overall group with CD and the overall group with UC compared with the HC
Yuksel et al. [6]	2016	Turkey	UC: active CD: active	UC: 36 CD: 25	64	Serum thiol (native and total)	native and total thiol were lower in UC e CD than in HC negative correlation between native thiol with EAI, CDAI, ESR and PCR in active CD and UC, when compared with HC
Neselioglu et al. [7]	2018	Turkey	UC active and inactive	UC active: 58 UC inactive: 20	58	Serum thiol (native and total)	native and total thiol were significantly lower in UC than in HC native and total thiol were significantly higher in UC in inactive than in active UC and near to those of HC significantly positive correlation: Thiol native x ALB in HC, UC Inactive and Active significantly negative correlation: Thiol native X PCR e Thiol native X ESR in HC, UC Inactive and Active significantly negative correlation:Truelove Witts X Thiol significantly negative correlation: TDH parameters and severity of UC significant negative correlations: severity of UC and native and total thiol homeostasis homeostasis parameters were significantly decreased in UC compared to HC There was no significant difference between UC inactive and HC Hemoglobin, hematocrit, and albumin levels were significantly low, ESR and CRP levels were significantly high in UC in active compared to HC
Bourgonje et al. [1]	2019	Netherlands	CD: inactive	CD: 51	27	Plasma free thiols albumin adjustedbel ow or above average	plasma free thiol albumin-adjusted were significantly lower in patients with CD inactive compared to HC The strongest association was observed between plasma free thiols albumin-adjusted and PCR CD with above-average free thiols had significantly lower CRP levels Univariable linear regression analyses confirmed that CRP and BMI were significantly associated inversely with plasma free thiol ALB adjusted multivariate linear BMI was independently associated with albumin-adjusted free thiols CD had significantly lower levels of Hb and ALB compared to HC CD had ESR and platelet counts were significantly increased compared to HC CD having solely colonic disease demonstrated markedly reduced plasma free thiol concentrations compared to patients with ileocolonic involvement Overall significant difference in albumin-adjusted plasma free thiols between different CD disease locations according to the Montreal classification HBI was not significantly different between patients with below or above average plasma free thiols Plasma free thiol negatively correlated with biomarkers of inflammation: CRP e IL-17A and the association with favorable disease status was further confirmed CD patients had significantly lower levels of hemoglobin and albumin whereas ESR and platelet counts were significantly increased compared to HC
Bourgonje et al. [2]	2019 a	Netherlands	UC: active CD: active	UC active:47 CD active: 31	50	Serum free thiols albumin adjusted	Albumin-adjusted serum free thiols are significantly reduced in UC and CD as compared to HC and strongly correlate with the degree of endoscopic disease activity Serum free thiol levels significantly negatively correlate to fecal calprotectin levels and may aid in diferentiating mild from moderate-to-severely active UC and CD as assessed by endoscopy Free thiols highly accurately discriminated between mild and moderate-to-severe disease activity, better than fecal calprotectin (FC) levels and this was maintained after crossvalidation and after adjustment for potentially confounding factors derived from univariable logistic regression analysis serum free thiols remained superior to fecal calprotectin levels in discriminating between mild and moderate-tosevere endoscopic disease activity Serum free thiol concentrations were significantly lower in both CD and UC as compared to HC Serum albumin- adjusted free thiol levels were significantly reduced in UC as compared to CD UC and CD with severe endoscopic disease activity had similar significantly lower levels of albumin adjusted serum free thiols compared to patients with mild disease activity Serum levels of albumin-adjusted free thiols significantly discriminated patients with mild disease activity from patients with moderate-to-severe disease activity - correlation between serum free thiols and endoscopic disease activity scores, both in the total IBD and separed CD and UC IBD cohort, serum concentrations of albumin-adjusted free thiols were significantly inversely associated with age, platelet counts and fecal calprotectin (FC) levels, and positively associated with duration of disease Association between serum free thiols and the SCCAI score for UC No significant correlation was found between serum free thiols with the HBI score for CD
Neubauer et al. [8]	2019	Poland	UC active and inactive CD active and inactive	UC active: 30 UC inactive: 41 CD active: 37 CD inactive: 10	57	Serum free thiols	Thiol concentrations were significantly lower in patients with both CD and UC, regardless the disease activity as compared with HC There were no significant differences between CD and UC or patients with active and inactive disease FT and TAS associations with IBD remained significant following the adjustment for age, sex, smoking status, and transferrin CD and UC patients with active disease, FT concentrations were inversely correlated with, respectively, CDAI and RI FT concentrations remained significantly lower in both CD and UC as compared to HC also following adjustment to albumin concentrations TAS was significantly reduced in both CD and UC patients as compared to HC, without significant differences between both disease phenotypes or with respect to the disease activity, but the inverse relation between TAS and CDAI or RI in IBD patients with active disease did not reach statistical significance In UC inversely thiol with TNF-a and the severity of bowel inflammation There is no relationship between antioxidants and disease duration in IBD or subgroups based on phenotypes or disease activity In CD FT were significantly lower in patients with anemia FT was significantly lower in patients treated with corticosteroids In active CD, FT was correlated with CRP, ESR, PLT and IL-6 in active CD, solely FT, inversely correlated with AOPP
Von Martels et al. [25]	2020	Netherlands	CD active	CD active: 70	0	Plasma free thiols albumin adjusted	Dietary riboflavin supplementation in CD patients for 3 weeks resulted in anti-inflammatory effects, reduction of clinical symptoms [HBI] and systemic oxidative stress, expressed by increased levels of thiols

*IBD* Inflammatory Bowel Disease, *UC* Ulcerative Colitis, *CD* Crohn’s Disease, *HC* Healthy Controls, *HBI* Harvey Bradshaw Index, *FT* Free Thiols, *TAS* Total Antioxidant Status, *IL-6* Interleukin 6, *BMI* Body Mass Index, *Hb* Hemoglobin, *RI* Rachmilewitz Index, *CDAI* Crohn’s Disease Activity Index, *AOPP* Advanced Oxidation Protein Products, *CRP* C-Reactive Protein, *ESR* Erythrocyte Sedimentation Rate, *TNF* Tumor Necrosis Factor

This systematic review included seven (7) analytical observational studies, case-control type [1, 2, 4–8] and only one (1) prospective clinical intervention study [25] that measured thiol levels in individuals with CD/UC with the aim of evaluating the potential association with oxidative stress and DII. Two studies [4, 7] evaluated only subjects with UC and two studies [1, 25] evaluated only subjects with CD. Only one study [1] evaluated only individuals in remission, one study [26] evaluated only individuals in active CD and two studies [5, 7] evaluated individuals with active and remitting IBD, comparing the results between individuals in different phases of the inflammatory disease. In all eight included studies [1, 2, 4–8, 25] there were changes in thiol levels in individuals with CD/UC and the authors inferred that there was oxidative stress associated with IBD.

### Type of measured thiol

Of the eight included studies in this systematic review, four [4–7] used the levels of native and/or total serum thiols as a marker of thiol oxidation; three studies [1, 2, 25] used plasma free thiol levels adjusted for albumin levels to assess systemic oxidative stress, and only one study [8] used plasma free thiol levels, but did not report whether there was a correction by plasma albumin levels, despite the attempt to obtain this methodological detail, by contacting the research group's electronic address. The lack of standardization of the type of thiol and the thiol dosage method limited the pooled analysis of the studies and made it impossible to compare the results in combined analyzes.

### Thiol expression and relationship with oxidative stress in inflammatory bowel disease

Baskol et al. [4] detected increased total thiol levels in subjects with UC compared to controls. Akinci et al. [5], when conducting the study with the largest number of individuals with IBD, included in this review, of both phenotypes, at different stages of the disease, found total thiol levels positively associated only with active CD. Yuksel et al. [6] identified a negative association between the reduction of total and native thiols in active CD and between native thiols in active UC, supported by a negative correlation between native thiol and EAI, CDAI, erythrocyte sedimentation rate (TSE) and C-reactive protein (CRP) in individuals with active CD and UC, when compared with healthy controls. Neselioglu et al. [7] found: I. negative association between: a: the levels of native and total thiols in individuals with active UC; b: native thiol and CRP; c: native thiol and TSE; d: thiol homeostasis and UC activity/severity; II. positive correlation between native thiol and albumin; III. lower total thiol levels in subjects with UC compared to healthy controls and IV. higher total and native thiol levels in subjects with UC in remission than subjects with active UC or healthy controls. From these results, these researchers related disease activity to thiol oxidation, and suggested of using thiol as a serum marker to assess activity and predict the severity of the disease course.

In 2019, the study conducted by Bourgonje et al. [1] was the precursor, among the included studies in this review, to measure the plasma concentrations of free thiols adjusting them to albumin, considering the characteristic of circulation of thiols in the human body [1, 9, 23, 24]. This group detected decreased levels of thiols in individuals with CD in remission compared to healthy controls, a negative correlation between plasma thiols and inflammation biomarkers, including CRP and IL-17A, enabling the correlation of subclinical CD activity to systemic oxidative stress. Another study, conducted by Bourgonje et al. [2] detected a strong correlation between plasma thiols and the degree of inflammatory disease activity evaluated endoscopically and a negative correlation between plasma thiol levels and fecal calprotectin (FC) levels in individuals with IBD, of both phenotypes, this time in activity, comparing them with healthy controls, allowing the discrimination, with high precision and in a significant way, of the degree of activity (mild, moderate or severe) of the disease, better than the FC. The Polish study conducted by Neubauer et al. [8] detected lower amounts of thiol in people with CD and UC, regardless of disease activity, when compared to healthy controls. Still, the thiol concentrations of individuals with CD and UC with active disease were inversely correlated with CDAI and Rachmilewitz Index (RI), respectively. However, this inverse relationship did not reach statistical significance. Based on these results, these authors suggested the use of plasma thiols as a therapeutic target to monitor IBD activity, as it is a minimally invasive strategy, presents an inverse correlation with the severity/severity of intestinal inflammation, and therapeutic modulation, through the administration of of antioxidants, considering that higher levels of plasma thiols would be associated with lower levels of inflammatory biomarkers and favorable systemic status and evolution in IBD.

It is important to consider that individuals with IBD treated with corticosteroids had lower thiol levels in the study conducted by Neubauer et al. [8]. Free thiol concentrations were decreased, mainly in the active CD, and were inversely related to inflammatory markers and oxidative stress, demonstrating depleted total antioxidant capacity, instrumentalizing these authors to conclude that the assessment of the total systemic antioxidant status can be useful in the evaluation not invasive of mucosal healing in individuals with IBD, and, additionally, that the assessment of serum thiol levels can provide relevant information about the adverse effects of corticosteroid therapy.

The most recent study included in this review was the one conducted by Von Martels et al. [25]. These researchers identified that there was no significant reduction in FC levels of the 70 patients with active CD after three weeks of riboflavin supplementation (100 mg daily). Still, thiol levels increased and clinical symptoms of CD decreased. These findings were attributed to the anti-inflammatory effects of riboflavin supplementation, which are associated with a reduction in oxidative stress, as measured by plasma levels of free thiols, which were increased.From the analysis of the included studies in this review, it was possible to identify relevant results related to systemic oxidative stress, measured by serum thiol levels, and IBD activity, and negative association with inflammatory markers. The findings of a strong correlation between the degree of endoscopic disease activity and a negative correlation between FC and serum thiols strengthen the justification for investigating the potential of thiols as a marker of oxidative stress in IBD.

### Methodological Quality and Risk of Bias

The assessment of the risk of bias of the selected studies is presented in Table 2 and Table 3. Eight studies were included in the assessment of the risk of bias.

The study conducted by Von Martels et al. [25] presents a domain (related to the outcome measure) with a high risk of bias, in the assessment by ROBINS I [17], for intervention studies, as proposed by the Cochrane Handbook [18]. Carrying out the intervention without a control group, measuring oxidative stress only in individuals with active DC, is a weakness in the study design, as it prevents accurate assessments, compromising the quality of the results.The other 7 studies included in this systematic review were observational studies, of the case-control type, and, for this reason, had the risk of bias and methodological quality evaluated by the instrument The Newcastle Ottawa Scale of Case-Control Studies [27], in agreement with the recommendation of the Cochrane Handbook [26]. In According to the methodological quality assessment proposed by this scale, it was observed that most studies presented more than 77.7% of adequacy in terms of quality, with percentages that varied between 66.6% and 88.8%. The issue that most contributed to the reduction in the methodological quality assessment and the increase in the risk of bias in these studies was the criteria for selecting controls, which were not described in some studies. None of the included studies assessed the outcome non-response rate, which determined the loss of points from all included studies in this review for this domain.

Based on the evaluation criteria of The Newcastle Ottawa Scale of Case-Control Studies [27], of the seven included studies in the review, two of them [1, 2] achieved the best evaluation and scored 8/9 stars, losing points for not presenting the outcome non-response rate assessment. Three studies [4, 7, 8] scored 7/9 stars; the studies by Baskol et al. [4] and Neubauer et al. [8] lost points due to the lack of characterization of the selection of controls and for not presenting the non-response rate in the studies. As for the study by Neselioglu et al. [7], the evaluation was reduced in the comparability of cases and controls and by the absence of evaluation of the non-response rate of the outcome.

The two studies [5, 6] rated as the lowest methodological quality in this review scored 6/9 stars, with 66.6% adequacy. The domains that determined the downgrading of the evaluation of these two studies were the selection and definition of controls [5] and the comparability of cases and controls [6]; these two studies also lost points for not evaluating the outcome non-response rate.

## Discussion

Findings from the individual studies included in this review suggest an association between disease activity and systemic oxidation, measured by serum thiol levels, a negative association with inflammatory markers and a strong correlation with the degree of endoscopic disease activity, justifying the authors' recommendation for the use of thiols as a parameter for CD and UC monitoring. On the other hand, it was not possible to perform a pooled analysis (meta-analysis) of the studies to assess the association between thiols and IBD activity, preventing statistical treatment to weight the results of individual studies. Rigorous systematic methods were employed to select the best quality studies currently available.

Thiols are organic compounds that are highly reactive and participate in immune regulation under physiological conditions [7, 22, 28], when the thiol in the reduced form is predominant in plasma. Oxidative stress, characterized by excessive production of ROS, intensifies redox reactions, modifying thiols and giving rise to oxidized products, such as thiol-disulfide, which are related to chronic diseases [10, 22, 29, 30]. Thiol/disulfide homeostasis can be measured by isolated levels or by indices that relate thiol and native and/or total disulfide. These parameters have been studied as markers of oxidative stress in inflammation situations [29, 30]. These concentrations may constitute biomarkers of oxidative stress and have special value in pathophysiological processes, such as UC and CD.

The correlation between CD and UC activity and the oxidative status of these individuals, as measured by thiol levels, and the association with clinical course and relapses, subclinical activity, the potential to monitor IBD activity/severity, and association with endoscopic activity has motivated investigations on the applicability of serum thiols in individuals affected by these intestinal conic inflammations, considering the significant advantage when compared to other markers traditionally used in clinical practice for the standard monitoring of IBD, mainly due to the minimally invasive character of this indicator.

The negative correlation between thiols and inflammation markers, such as CRP and ESR, and with indices of UC and CD activity, such as CDAI and RI, are findings that support the hypothesis of oxidation associated with active IBD and that were identified in most studies more robust [1, 2, 7, 8] included in this review. On the other hand, the positive association detected between thiols and albumin is the expression of a favorable behavior for a good prognosis in IBD, which can be verified in these studies, which strengthens the association of thiols as a biomarker in IBD.Two studies could not be included in this review [10, 31] because they did not meet the eligibility criteria, considering the different characteristics of the evaluated species, preventing the extrapolation and applicability of the results, they also identified an association between oxidative stress and DII, attributing an important role to biomarkers in monitoring CD and UC.

The impossibility of performing meta-analyses based on individual studies is highlighted, due to the lack of standardization of the variables in these studies, which limits the certainty of the results and the extrapolation to clinical practice.

### Limitations

It is essential to highlight the limitations of this review, considering that it was not possible to verify the pooled assessment of the studies, due to the lack of combinable studies. There was no standardization of the type of thiol measured and the use of different methods to measure thiol levels in CD patients, UC and controls prevented associations between studies and impossibility of performing a meta-analysis determines a reduction in the strength of the evidence and prevents a more robust assessment of the certainty of the results.

All included studies [1, 2, 4–8, 25] mentioned the use of spectrophotometric reading to measure thiols, however, only five studies [1, 2, 4, 8, 25] mentioned the use of Ellman's reagent in the description of the method used, which may have interfered with the results found, constituting a difficulty for the reproducibility and reliability of the studies.

The absence of standardization regarding I. the IBD phenotype; II. the evaluation of individuals in different phases (remission/activity) of the inflammatory disease; III. to the different types of thiol (native/total/free thiol) measured, and IV. the different techniques (types of reagents/albumin correction) used to measure thiols were factors that contributed to increasing the heterogeneity of information among the included studies in this review, interfering with the systematization, data analysis and consolidation of consistent and reliable evidence on the relationship between thiols and IBD.

Yet the absence of controls in the study weakens the reliability of the results available in the study [26]

The included studies varied in sample size, but all included a small number of individuals with IBD. The smallest study [4] evaluated 30 subjects with UC compared to 30 healthy controls, and the largest study was conducted by Akinci et al. [5], evaluating 177 individuals with IBD (CD: n =118/UC: n= 59), comparing them with 30 healthy controls. The small samples of the included studies may have reduced the power to detect positive associations between exposure and outcome.

The evaluation of the methodological quality of the included studies showed that the lack of characterization of the selection of controls, of comparability of cases and controls and the non-response rate in the studies were the factors that contributed to the lowering of the methodological quality of the included studies and determined low confidence in the results found. Additionally, the heterogeneity, wide confidence intervals and inconsistencies of the studies are important factors for the increased risk of bias in the studies and low certainty of the evidence of the evaluated outcomes, reducing the safety for decision-making in clinical practice, and justifying the performance of new verifications.

The limitations inherent to this systematic review were overcome by the methodological rigor applied in all stages of this work, carried out by two independent researchers, and through the application of instruments to assess the risk of bias; estimates of effectiveness and safety in observational studies, in addition to the assessment of the certainty of the evidence.

No other studies or reviews were identified evaluating the association of thiols and IBD that disagreed with the results of the included studies in this systematic review.

We recommend carrying out better-designed and controlled observational studies, including individuals of both phenotypes and at different stages of IBD, in different groups, involving a larger number of participants, standardization of the technique for measuring serum thiols, to confirm whether thiols may be a good parameter for monitoring the clinical course of these intestinal diseases.

## Conclusions

The association between increased oxidation associated with active disease needs to be confirmed, in order to allow the applicability of the use of thiols for the assessment of disease activity and severity, as well as the response to therapy, considering the practicality, low cost and minimally invasive nature of the method, which characterizes it as a promising strategy for the management of UC and CD. Additionally, thiols can provide relevant information about the adverse effects of nutritional and drug therapy used to treat these individuals, and provide important information for early clinical and nutritional interventions.

Further studies are needed to confirm the efficacy of thiols and the potential for association to predict CD and UC activity, so that the degree of clinical applicability for the approach to IBD can be established.

## Supplementary Information

**Additional file 1: Table S1.** Studies Data.

## Data Availability

The dataset supporting the conclusions of this article is included as a supplementary file.

## Abbreviations

IBD: Inflammatory bowel disease
UC: Ulcerative colitis
CD: Crohn’s disease
ROS: Reactive oxygen species
PRISMA: Preferred reporting items for systematic reviews and meta-analyses
FC: Fecal calprotectin
HC: Healthy controls
HBI: Harvey bradshaw index
FT: Free thiols
TAS: Total antioxidant status
IL-6: Interleukin 6
BMI: Body mass index
Hb: Hemoglobin
RI: Rachmilewitz index
CDAI: Crohn’s disease activity index
AOPP: Advanced oxidation protein products
CRP: C-Reactive protein
ESR: Erythrocyte sedimentation rate
TNF: Tumor necrosis factor
